# Deep Vein Thrombosis Prevention in Acute Ischemic Stroke Patients with Lower Limb Paralysis: A Narrative Review

**DOI:** 10.3390/jcm15062091

**Published:** 2026-03-10

**Authors:** Jianyu Peng, Shiyan Long, Ling Feng

**Affiliations:** 1 Department of Neurology, West China Hospital, Sichuan University/West China School of Nursing, Sichuan University, Chengdu 610041, China; 15196788875@163.com (J.P.); 13880602153@163.com (S.L.); 2 Department of Neurology, West China Tianfu Hospital, Sichuan University, Chengdu 610200, China

**Keywords:** acute ischemic stroke, deep vein thrombosis, lower limb paralysis, prevention, thromboprophylaxis, immunothrombosis

## Abstract

Patients with lower limb paralysis following acute ischemic stroke (AIS) are at a markedly increased risk of deep vein thrombosis (DVT), which may lead to pulmonary embolism and substantially higher mortality and disability. This review comprehensively reviews studies from the past decade on the epidemiology, pathophysiology, and prevention of DVT in AIS patients with lower limb paralysis. The pathogenesis of DVT in this population is multifactorial, involving venous stasis due to immobility, stroke-induced hypercoagulability, endothelial dysfunction, neutrophil extracellular trap-mediated immunothrombosis, and autonomic dysregulation. Effective prevention requires individualized risk stratification, integrating clinical assessment, biomarkers, and imaging tools. Current prophylactic strategies include pharmacological anticoagulation (primarily low-molecular-weight heparin), mechanical interventions (such as intermittent pneumatic compression), and early mobilization and rehabilitation. While combined approaches have demonstrated significant benefits, challenges remain regarding the timing of anticoagulation, balancing bleeding risks, extended thromboprophylaxis, and novel immunothrombosis targets. Future research should focus on personalized prevention protocols, the application of artificial intelligence-based predictive models, and innovative therapies targeting endothelial injury and immune-mediated thrombosis, aiming to improve thromboprophylaxis and overall outcomes in this high-risk population.

## 1. Introduction

Acute ischemic stroke (AIS) remains a leading cause of death and long-term disability worldwide, accounting for substantial healthcare and socioeconomic burdens [1,2]. Despite major advances in acute stroke management, particularly intravenous thrombolysis and endovascular thrombectomy, stroke-related complications continue to significantly affect patient outcomes [3]. Among these complications, deep vein thrombosis (DVT) is one of the most common yet frequently under-recognized conditions [4], especially in patients with severe neurological deficits such as lower limb paralysis.

The development of DVT following AIS is clinically significant not only because of local venous complications, including limb swelling, pain, and delayed rehabilitation, but also due to its potential progression to pulmonary embolism (PE), a life-threatening condition associated with increased mortality [5,6]. Epidemiological studies consistently demonstrate that stroke patients may have a higher risk of venous thromboembolism (VTE) compared with the general hospitalized population, with immobilized patients representing the highest-risk subgroup.

Lower limb paralysis is a hallmark of moderate-to-severe AIS and serves as a critical determinant of venous stasis [7,8]. The loss of voluntary muscle contraction compromises the calf muscle pump mechanism, leading to impaired venous return and blood pooling in the deep veins of the lower extremities [9,10]. This pathophysiological alteration fulfills a central component of Virchow’s triad and creates a permissive environment for thrombogenesis. Importantly, immobility alone does not fully explain the heightened thrombotic risk observed after stroke. Increasing evidence suggests that AIS induces a systemic prothrombotic state characterized by endothelial dysfunction, inflammatory activation, and dysregulation of coagulation and fibrinolytic pathways [11,12].

Over the past decade, research has expanded our understanding of stroke-associated DVT beyond traditional concepts of immobility. Stroke-induced immunothrombosis, neutrophil extracellular trap (NET) formation, endothelial activation, and autonomic dysregulation have emerged as critical contributors to venous thrombosis [11]. Concurrently, preventive strategies have evolved from routine anticoagulation toward integrated, individualized approaches combining pharmacological, mechanical, and rehabilitative interventions [13,14].

Despite these advances, optimal DVT prevention in AIS patients with lower limb paralysis remains challenging. Clinical decisions must balance the benefits of anticoagulation against the risk of hemorrhagic transformation, particularly in the acute phase of ischemic stroke. Furthermore, heterogeneity in patient characteristics, stroke severity, and comorbidities necessitates refined risk stratification tools and personalized prophylactic regimens.

**Figure 1:**
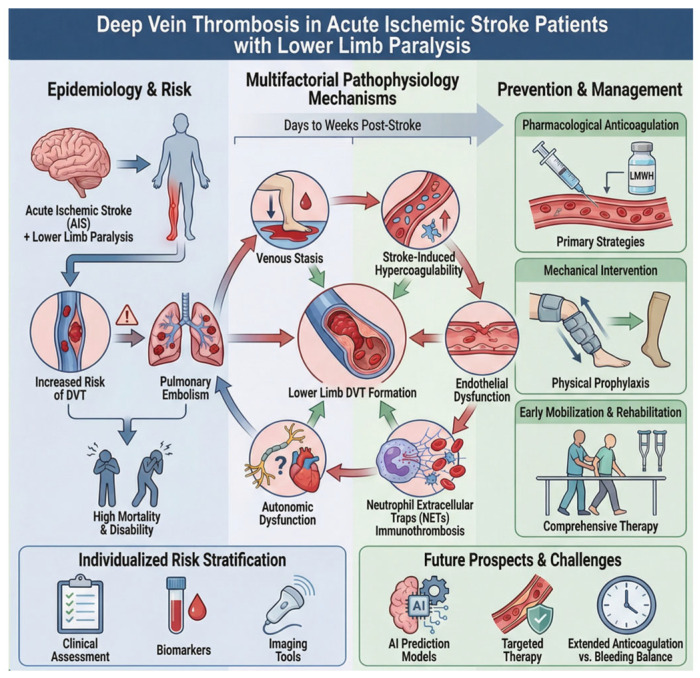
Overview of DVT in acute ischemic stroke (AIS) patients with lower limb paralysis.

This review aims to provide a comprehensive synthesis of current evidence regarding the epidemiology, pathophysiological mechanisms, and preventive strategies for DVT in AIS patients with lower limb paralysis (Figure 1). By integrating findings from clinical and preclinical studies published over the past decade, we seek to identify knowledge gaps, highlight emerging therapeutic targets, and propose future directions for improving DVT prevention and patient outcomes.

## 2. Search Strategy and Study Selection

A comprehensive literature search was performed in PubMed, Embase, and Web of Science for articles published between January 2015 and October 2025. The search strategy combined terms related to acute ischemic stroke, lower limb paralysis, deep vein thrombosis, and preventive interventions. We included original studies, systematic reviews, and guidelines focusing on DVT prevention in AIS patients with lower limb paralysis, prioritizing evidence from high-quality studies while noting when data were extrapolated from non-stroke populations.

## 3. Epidemiology of Deep Vein Thrombosis in Acute Ischemic Stroke with Lower Limb Paralysis

The reported incidence of DVT in AIS varies widely depending on study design, diagnostic modality, timing of assessment, and patient population. Earlier studies relying on clinical symptoms alone underestimated the true burden of DVT, as a substantial proportion of cases, particularly distal DVT, remain asymptomatic. With the increased application of conventional duplex ultrasound examinations, contemporary research indicates that the detection rate of deep vein thrombosis in patients has risen, thereby effectively enhancing the quality of research [15,16,17].

Studies have demonstrated that lower limb paralysis is a major independent predictor of post-stroke DVT [18,19]. In a large cohort study involving 679 acute stroke patients, the overall incidence of DVT was 21.1% in ischemic stroke, with intermuscular and distal calf veins being the most commonly affected sites [19]. Patients with hemiplegia or paraplegia exhibited significantly higher DVT rates than those with mild motor deficits. Notably, hemorrhagic stroke patients demonstrated an even higher incidence, underscoring the complex interplay between stroke subtype and thrombotic risk. These findings from hemorrhagic stroke populations may not be directly generalizable to AIS due to differences in coagulopathy and management protocols.

The timing of DVT occurrence after AIS is another critical consideration. Most events occur within the first weeks following stroke onset, coinciding with the period of maximal immobility and inflammatory response. However, studies have shown that the risk persists beyond hospital discharge, particularly in patients with prolonged paralysis, limited rehabilitation participation, or additional risk factors such as infection or malignancy [20,21]. This prolonged vulnerability supports the rationale for extended-duration thromboprophylaxis in selected high-risk patients [22,23,24].

Geographic and healthcare system differences further influence DVT epidemiology [25,26]. Studies from Asia report DVT incidences comparable to or slightly lower than those observed in Western populations, although differences in screening practices and prophylaxis protocols complicate direct comparisons [27]. Importantly, disaster-related immobilization studies, such as those conducted after major earthquakes in Japan, provide compelling evidence that immobility alone can precipitate DVT, with reported incidences of 10–30% within one to two weeks among individuals confined to shelters [28]. These findings offer valuable insights into the mechanisms underlying DVT in immobilized stroke patients.

From a prognostic standpoint, the occurrence of DVT after AIS is associated with worse functional outcomes and increased mortality [29]. Patients who develop DVT are more likely to experience delayed rehabilitation, prolonged hospitalization, and recurrent medical complications [30]. Pulmonary embolism remains a leading cause of sudden death in stroke patients [31], emphasizing the need for early identification and effective prevention strategies.

## 4. Pathophysiological Mechanisms of DVT in AIS with Lower Limb Paralysis

### 4.1. Venous Stasis and Failure of the Neuromuscular Pump

Venous stasis represents the most immediate and intuitive mechanism linking lower limb paralysis to DVT formation [32]. Under physiological conditions, rhythmic contraction of calf and thigh muscles propels venous blood toward the heart, aided by unidirectional venous valves [33,34,35]. Lower limb paralysis disrupts this neuromuscular pump, resulting in reduced venous flow velocity, increased venous pressure, and blood pooling in deep veins [36]. Prolonged venous stasis leads to hypoxia within the venous wall, promoting endothelial activation and local inflammatory responses that further predispose to thrombosis [37,38]. Experimental models show that sustained stasis alters venous valve geometry, causing leaflet deformation and microinjury, which serve as niduses for thrombus initiation [39].

Clinical observations reinforce the importance of venous stasis. Bed rest exceeding three days has been identified as an independent risk factor for post-stroke DVT, even after adjusting for age and comorbidities [40]. Conversely, restoration of muscle tone and early mobilization significantly reduce thrombotic risk [41], highlighting the dynamic and potentially reversible nature of stasis-induced thrombogenesis.

### 4.2. Stroke-Induced Hypercoagulable State

Beyond mechanical stasis, AIS induces a systemic hypercoagulable state that amplifies thrombosis risk [11]. Cerebral ischemia triggers a cascade of inflammatory and neurohumoral responses that extend well beyond the central nervous system [42,43]. Elevated levels of procoagulant factors such as fibrinogen, factor VIII, and von Willebrand factor have been documented in the acute phase of ischemic stroke [44,45]. Simultaneously, natural anticoagulant pathways involving protein C, protein S, and antithrombin may be suppressed [46,47].

Platelet activation is another hallmark of stroke-associated hypercoagulability. Increased platelet reactivity and aggregation have been observed following AIS, driven in part by inflammatory mediators and endothelial activation [48]. These changes contribute not only to arterial thrombosis but also to venous clot formation, particularly in the context of venous stasis. Systemic inflammation plays a central role in maintaining this hypercoagulable milieu [49]. Elevated circulating cytokines, including interleukin-1β and tumor necrosis factor-α, enhance tissue factor expression on endothelial cells and monocytes, tipping the hemostatic balance toward thrombosis [50,51]. Reduced fibrinolytic activity, mediated by increased plasminogen activator inhibitor-1 (PAI-1), further impairs clot resolution. Together, these alterations establish a prothrombotic state that persists for days to weeks after AIS, explaining why DVT risk remains elevated even after partial mobilization. Importantly, this hypercoagulability interacts synergistically with venous stasis and endothelial injury, reinforcing the multifactorial nature of stroke-associated DVT.

### 4.3. Vascular Endothelial Dysfunction and Stroke-Associated Thrombogenesis

Vascular endothelial cells play a central role in maintaining vascular homeostasis by regulating anticoagulant, antiplatelet, and fibrinolytic functions [52,53,54]. Following acute ischemic stroke, endothelial integrity is profoundly disrupted, both locally within the cerebral vasculature and systemically throughout the circulation [55,56,57]. This widespread endothelial dysfunction represents a critical mechanistic link between cerebral ischemia and peripheral venous thrombosis.

Ischemia–reperfusion injury after AIS leads to excessive production of reactive oxygen species (ROS), mitochondrial dysfunction, and activation of inflammatory signaling pathways within endothelial cells [58,59,60]. These processes result in loss of endothelial barrier function, increased permeability, and a phenotypic shift from an antithrombotic to a prothrombotic state. Activated endothelial cells upregulate adhesion molecules such as E-selectin, intercellular adhesion molecule-1 (ICAM-1), and vascular cell adhesion molecule-1 (VCAM-1), facilitating leukocyte rolling, adhesion, and transmigration [61,62]. This leukocyte–endothelial interaction is a critical early step in thrombus formation.

Endothelial cells also actively contribute to coagulation through the release of prothrombotic mediators. Increased expression of tissue factor initiates the extrinsic coagulation cascade, while enhanced secretion of von Willebrand factor (vWF) promotes platelet adhesion and aggregation [63]. Notably, elevated circulating vWF levels have been consistently observed in AIS patients and are associated with worse outcomes and higher thrombotic risk [64,65]. The imbalance between vWF and its cleaving protease ADAMTS13 further exacerbates thrombogenicity, particularly in conditions of systemic inflammation [66,67].

Recent experimental studies have identified specific intracellular signaling pathways linking inflammatory cytokines to endothelial thrombogenic responses. The Gab2–MALT1 signaling axis has emerged as a key regulator of IL-1β-induced endothelial activation [68,69]. Activation of this pathway promotes Weibel–Palade body exocytosis, resulting in rapid surface expression of P-selectin and release of vWF. Inhibition of MALT1 protease activity markedly reduced endothelial inflammation and neutrophil recruitment [70,71], potentially exerting a mitigating effect on venous thrombosis. These findings indicate that endothelial cells are not passive bystanders but active participants in the process of stroke-associated venous thrombosis.

Moreover, endothelial cell death modalities such as pyroptosis and ferroptosis have been implicated in vascular injury after stroke [72,73]. Oxidative stress-induced lipid peroxidation and inflammasome activation contribute to endothelial dysfunction, amplifying inflammatory and procoagulant signaling [74]. Therapeutic agents targeting endothelial oxidative stress and inflammatory pathways, including peroxisome proliferator-activated receptor gamma (PPARγ) agonists and endothelial nitric oxide synthase (eNOS) activators, have demonstrated protective effects in preclinical deep vein thrombosis models, indicating their potential translational medical significance [75,76].

Clinical Implications: The recognition of endothelial dysfunction as a central driver of stroke-associated thrombosis has direct clinical relevance. Elevated circulating levels of von Willebrand factor (vWF) and soluble adhesion molecules (e.g., ICAM-1, VCAM-1) may serve as useful biomarkers for identifying AIS patients at heightened risk of DVT, potentially guiding more aggressive prophylactic strategies. Furthermore, therapeutic agents targeting endothelial oxidative stress and inflammatory pathways, such as peroxisome proliferator-activated receptor gamma (PPARγ) agonists and endothelial nitric oxide synthase (eNOS) activators, have demonstrated protective effects in preclinical DVT models. Although not yet incorporated into routine clinical practice, these agents represent promising avenues for future adjunctive thromboprophylaxis, particularly in patients with contraindications to anticoagulation.

### 4.4. Neutrophil Extracellular Traps and Immunothrombosis

Over the past decade, the concept of immunothrombosis has fundamentally reshaped our understanding of venous thromboembolism. Rather than being driven solely by coagulation abnormalities, thrombosis is now recognized as an immune-mediated process involving complex interactions between leukocytes, platelets, and the endothelium [77]. Neutrophil extracellular traps (NETs) have emerged as central mediators of this process in both arterial and venous thrombosis [78,79]. NETs are web-like structures composed of decondensed chromatin decorated with histones, myeloperoxidase, neutrophil elastase, and other proteolytic enzymes [80]. While NET formation (NETosis) serves a physiological role in host defense, excessive or dysregulated NET release promotes thrombosis by providing a scaffold for platelet adhesion, fibrin deposition, and coagulation factor activation [81]. NETs directly activate factor XII and enhance thrombin generation, thereby linking innate immunity to the coagulation cascade [82]. Acute ischemic stroke is a potent trigger of systemic NETosis [83]. Circulating markers of NET formation, including cell-free DNA, citrullinated histone H3, and myeloperoxidase–DNA complexes, are significantly elevated in AIS patients and correlate with stroke severity [84]. Experimental stroke models demonstrate that cerebral ischemia induces neutrophil activation not only within the brain but also in peripheral vascular beds, including the venous system [85].

In the context of lower limb paralysis, NET-mediated thrombosis is further facilitated by venous stasis and endothelial activation. Stagnant blood flow prolongs the interaction time between neutrophils, platelets, and the vessel wall, creating favorable conditions for NET deposition [86]. Endothelial-derived P-selectin and vWF promote neutrophil tethering, while inflammatory cytokines such as IL-1β and TNF-α amplify NET release [87]. Once formed, NETs stabilize venous thrombi and render them more resistant to endogenous fibrinolysis.

Importantly, experimental studies suggest that targeting NETs may represent a novel strategy for DVT prevention [88]. DNase I treatment, which degrades extracellular DNA, significantly reduces thrombus size in murine venous thrombosis models [89]. Similarly, inhibition of peptidylarginine deiminase 4 (PAD4), a key enzyme required for NET formation, attenuates thrombosis without impairing hemostasis [90,91]. Although these approaches remain investigational, they provide compelling proof-of-concept for immune-targeted thromboprophylaxis in stroke patients.

Clinical Implications: The emerging concept of immunothrombosis highlights the critical role of inflammation in DVT pathogenesis and opens new therapeutic possibilities. Neutrophil extracellular traps (NETs) and their components (e.g., cell-free DNA, myeloperoxidase) are detectable in plasma and may serve as novel biomarkers for thrombotic risk stratification in AIS patients. Pharmacological strategies targeting NET formation or degradation, including DNase I, PAD4 inhibitors, and agents that disrupt the Gab2-MALT1 signaling pathway, are currently under investigation and may eventually complement conventional anticoagulation. From a practical standpoint, minimizing systemic inflammation through early infection control, adequate hydration, and prompt management of comorbidities represents an immediately actionable approach to reducing immunothrombotic risk in the stroke unit.

### 4.5. Autonomic Nervous System Dysregulation and Venous Hemodynamics

The autonomic nervous system (ANS) plays a critical yet underappreciated role in regulating vascular tone and venous capacitance. Acute ischemic stroke frequently disrupts central autonomic control, resulting in sympathetic–parasympathetic imbalance [92,93]. This dysregulation has profound implications for venous hemodynamics and thrombosis risk.

Sympathetic activation following stroke can lead to abnormal vasoconstriction or vasodilation in peripheral veins, altering venous capacitance and pressure [94,95]. Impaired sympathetic regulation of venous smooth muscle tone reduces the efficiency of venous return, particularly in the lower extremities [10]. This effect is compounded by lower limb paralysis, which eliminates the compensatory action of the skeletal muscle pump.

Clinical studies have demonstrated that stroke patients with autonomic dysfunction exhibit greater variability in blood pressure and heart rate, as well as impaired microcirculatory perfusion [96]. These hemodynamic disturbances contribute to endothelial shear stress abnormalities, which promote endothelial activation and prothrombotic signaling. In animal models, physical interventions regulating autonomic nervous system activity, such as intermittent pneumatic compression combined with local heat therapy, have been demonstrated to enhance venous blood flow velocity, mitigate venous valve deformation, and reduce thrombotic risk [97].

These findings underscore the importance of considering neurovascular interactions in the pathogenesis of DVT after stroke. They also provide a physiological rationale for mechanical prophylactic strategies that aim to restore venous flow dynamics in immobilized patients.

### 4.6. Systemic Modifiers: Infection, Dehydration, and COVID-19

Systemic conditions frequently encountered in AIS patients further modulate thrombosis risk. Post-stroke infections, particularly pneumonia and urinary tract infections, are common complications that markedly increase the likelihood of DVT [98]. Inflammatory responses associated with infection amplify cytokine release, endothelial activation, and coagulation pathway activation. Retrospective studies have identified pulmonary infection as a strong independent predictor of post-stroke DVT, with odds ratios exceeding four in some cohorts [99].

Dehydration is another critical yet often overlooked contributor to hypercoagulability in AIS [100]. Dysphagia, reduced consciousness, and inadequate fluid intake lead to hemoconcentration and increased blood viscosity, thereby promoting venous stasis and clot formation [101]. Maintaining adequate hydration is therefore a simple but essential component of comprehensive DVT prevention.

The COVID-19 pandemic has added a new layer of complexity to thrombosis management in stroke patients. SARS-CoV-2 infection induces a profound prothrombotic state characterized by endothelialitis, platelet activation, and widespread immunothrombosis [102]. Critically ill COVID-19 patients exhibit exceptionally high rates of venous thromboembolism, and AIS patients with concurrent COVID-19 infection appear to face compounded thrombotic risk [103,104]. Endothelial injury, NET overproduction, and dysregulated coagulation converge in this setting, highlighting shared mechanisms between viral infection and stroke-associated thrombosis. However, these findings originate from COVID-19 cohorts and may not be fully generalizable to the routine AIS population without concomitant infections.

### 4.7. Genetic and Individual Susceptibility Factors

Although acquired risk factors predominate in AIS-associated DVT, genetic predisposition may influence individual susceptibility [105,106]. Inherited thrombophilias such as protein C deficiency, protein S deficiency, antithrombin deficiency, and factor V Leiden mutation are relatively rare but can markedly increase venous thrombotic risk [107,108]. These conditions impair endogenous anticoagulant pathways, leading to unchecked thrombin generation.

Case reports and small series have documented severe or recurrent DVT in young stroke patients later diagnosed with inherited thrombophilia [109,110,111]. While routine genetic screening is not recommended for all AIS patients, consideration of underlying thrombophilia is warranted in individuals with unexplained, recurrent, or extensive thrombotic events, particularly when occurring at a young age or in the absence of traditional risk factors. Recognition of genetic susceptibility may influence decisions regarding the intensity and duration of anticoagulation.

## 5. Risk Stratification and Predictive Models for DVT in AIS with Lower Limb Paralysis

Effective prevention of deep vein thrombosis in acute ischemic stroke patients with lower limb paralysis requires accurate identification of individuals at the highest risk. However, risk stratification in this population remains challenging due to the heterogeneity of stroke severity, comorbidities, and dynamic changes in coagulation and inflammatory status. Over the past decade, increasing efforts have been directed toward developing predictive models that integrate clinical, laboratory, and imaging variables to improve individualized risk assessment. Table 1 summarizes the key DVT risk factors specific to AIS patients with lower limb paralysis, categorized by patient-related, stroke-related, and complication-related domains.

### 5.1. Clinical Risk Factors

Traditional clinical risk factors form the foundation of DVT risk assessment in AIS patients. Advanced age, severity of neurological deficit, and degree of immobility are consistently identified as strong predictors [112,113]. Patients with higher National Institutes of Health Stroke Scale (NIHSS) scores, reflecting more severe motor impairment and reduced consciousness, exhibit substantially higher DVT incidence [114,115]. Lower limb paralysis, particularly complete hemiplegia or paraplegia, is among the most robust predictors, underscoring the central role of venous stasis [7].

Prolonged bed rest is another well-established risk factor. Observational studies demonstrate that immobilization exceeding three days independently increases DVT risk, even after adjustment for age and comorbidities [40]. Additional clinical factors include a prior history of venous thromboembolism, active malignancy, obesity, heart failure, and acute infections such as pneumonia [116]. These clinical variables are easily assessed at the bedside and form the basis of initial risk stratification.

### 5.2. Laboratory Biomarkers

Laboratory biomarkers provide objective insights into the underlying prothrombotic state. Among these, D-dimer remains the most widely studied marker [117,118,119]. Elevated D-dimer levels are common after AIS due to cerebral ischemia and inflammation; however, markedly increased or rising D-dimer levels have been associated with subsequent DVT development [8]. D-dimer testing is routinely available in most clinical laboratories and can be serially monitored.

Inflammatory markers such as C-reactive protein (CRP), interleukin-6, and neutrophil-to-lymphocyte ratio (NLR) have also been linked to an increased DVT risk [120]. These markers reflect the intensity of systemic inflammation and immunothrombosis, processes increasingly recognized as central to stroke-associated venous thrombosis. Elevated fibrinogen levels and increased factor VIII activity further indicate a hypercoagulable state and have been associated with poor clinical outcomes [121].

While CRP and NLR are routinely measured in many institutions, their role in DVT prediction specifically remains adjunctive. Markers of endothelial dysfunction, including von Willebrand factor and soluble P-selectin, represent emerging candidates for risk stratification [122,123]. Elevated vWF levels correlate with stroke severity, endothelial activation, and thrombotic events. However, these assays are not yet routinely available in most clinical laboratories and should be considered experimental tools requiring further validation.

### 5.3. Imaging-Based Risk Assessment

Imaging modalities play a complementary role in DVT risk stratification [124]. Baseline lower extremity duplex ultrasonography can identify asymptomatic distal thrombosis, which may progress proximally if untreated. However, routine screening of all AIS patients remains controversial due to resource limitations and variable cost-effectiveness. Advanced neuroimaging may also provide indirect risk indicators. Large infarct volume, involvement of motor pathways, and severe white matter damage are associated with prolonged immobility and autonomic dysfunction, indirectly increasing DVT risk [125]. While these neuroimaging features are routinely assessed for clinical management, their integration into formal DVT prediction models remains investigational.

### 5.4. Nomograms and Multivariable Predictive Models

To overcome the limitations of single-factor assessment, several studies have developed multivariable predictive models and nomograms for post-stroke DVT [126,127,128]. These tools typically integrate age, stroke severity (NIHSS score), duration of immobilization, laboratory markers (e.g., D-dimer), and comorbidities.

Recent nomogram-based studies demonstrate moderate-to-high predictive accuracy in derivation cohorts [129]. However, it is important to note that most of these models have undergone only internal validation; few have been externally validated in independent populations or prospectively tested. Therefore, while promising, their use in routine clinical practice is not yet widespread and should be considered investigational.

### 5.5. Artificial Intelligence and Machine Learning Approaches

Advances in artificial intelligence (AI) and machine learning have opened new avenues for DVT risk prediction in AIS [130,131,132]. Machine learning algorithms can process high-dimensional data, including clinical variables, laboratory trends, and imaging features, to identify complex, nonlinear associations that may be overlooked by traditional statistical methods. Recent studies applying random forest, support vector machine, and neural network models have demonstrated superior predictive performance compared with conventional logistic regression models [133]. Despite these promising results, these approaches remain largely investigational. They require further external validation in large, multicenter cohorts, integration with electronic health record systems, and prospective testing before they can be recommended for routine clinical implementation.

## 6. Pharmacological Prophylaxis of DVT in AIS with Lower Limb Paralysis

Pharmacological thromboprophylaxis remains the cornerstone of DVT prevention in acute ischemic stroke patients with lower limb paralysis. However, optimal agent selection, timing, and duration of therapy require careful consideration of both thrombotic and hemorrhagic risks. Throughout this section, we explicitly distinguish between evidence derived from stroke-specific populations and data extrapolated from studies of general medical inpatients, highlighting areas where AIS-focused randomized trials are lacking.

### 6.1. Low-Molecular-Weight Heparin Versus Unfractionated Heparin

Low-molecular-weight heparin (LMWH) is generally preferred over unfractionated heparin (UFH) for DVT prophylaxis in AIS [134]. Multiple randomized trials and meta-analyses have demonstrated that LMWH is more effective in reducing the incidence of DVT and pulmonary embolism [135]. Notably, while some individual trials included mixed populations of medically ill patients, stroke-specific meta-analyses have consistently confirmed the superiority of LMWH over UFH in AIS cohorts. LMWH offers several practical advantages, including predictable pharmacokinetics, once- or twice-daily dosing, and a lower risk of heparin-induced thrombocytopenia. Enoxaparin is the most commonly used LMWH in stroke units worldwide [136]. In contrast, UFH requires frequent dosing and laboratory monitoring, which may increase nursing workload and variability in anticoagulant effect.

### 6.2. Timing of Anticoagulation After Thrombolysis or Thrombectomy

The timing of pharmacological prophylaxis initiation is critical in AIS, particularly in patients receiving reperfusion therapies [137,138]. Following intravenous thrombolysis with tissue plasminogen activator (tPA), anticoagulation is typically deferred until follow-up neuroimaging confirms the absence of intracranial hemorrhage [139,140]. In patients undergoing mechanical thrombectomy, timing decisions are influenced by infarct size, hemorrhagic risk, and post-procedural stability. Although early anticoagulation may reduce DVT risk, premature initiation increases the risk of hemorrhagic transformation. Therefore, individualized assessment remains essential.

### 6.3. Standard In-Hospital Prophylaxis

Standard in-hospital thromboprophylaxis with LMWH typically lasts throughout the acute hospitalization period (generally 6–14 days) [141]. This practice is supported by stroke-specific evidence from multiple randomized trials demonstrating significant DVT risk reduction with LMWH compared to placebo or UFH. Current guidelines uniformly recommend pharmacological prophylaxis for immobilized AIS patients in the absence of contraindications.

### 6.4. Extended Thromboprophylaxis

A meta-analysis of four randomized trials (APEX, EXCLAIM, MAGELLAN, and MARINER) including 4330 AIS patients demonstrated that extended thromboprophylaxis for 4–5 weeks reduced VTE risk compared to standard-duration prophylaxis (RR 0.67; 13 fewer per 1000) without a significant increase in major bleeding (RR 1.10; 1 more per 1000) [24]. However, these findings derive from subgroup analyses of trials in medically ill populations, not from AIS-dedicated studies, and patients on dual antiplatelets or thrombolysis were excluded, limiting generalizability [24].

Based on the available extrapolated evidence, AIS patients with persistent lower limb paralysis, severe immobility (e.g., NIHSS motor score ≥ 2 for leg beyond 7 days), active cancer, or markedly elevated D-dimer levels (>3× upper limit of normal at day 7) may benefit most from extended prophylaxis [24,142]. Suggested duration ranges from 4 to 6 weeks post-discharge, using regimens evaluated in the trials (e.g., rivaroxaban 10 mg daily, enoxaparin 40 mg daily). These recommendations are based on limited evidence and should be individualized with careful assessment of bleeding risk. AIS-specific randomized trials are urgently needed to establish definitive criteria and optimal regimens.

### 6.5. Direct Oral Anticoagulants: Emerging Evidence and Controversies

Direct oral anticoagulants (DOACs), including factor Xa inhibitors and direct thrombin inhibitors, have transformed long-term VTE prevention in other clinical settings [143,144]. Their role in primary DVT prophylaxis after AIS, however, remains under investigation. Trials in medically ill patients have demonstrated that agents such as rivaroxaban and betrixaban reduce post-discharge VTE risk when used for extended prophylaxis [145,146]. While these findings are encouraging, stroke-specific evidence is limited. Concerns regarding intracranial bleeding, optimal dosing, and patient selection have precluded routine DOAC use in the acute stroke setting. Future randomized trials focusing on high-risk AIS patients are needed to clarify their safety and efficacy.

### 6.6. Bleeding Risk and Hemorrhagic Transformation

A central challenge in pharmacological prophylaxis is balancing thrombosis prevention against bleeding risk. Hemorrhagic transformation is a feared complication of AIS, particularly in patients with large infarcts, uncontrolled hypertension, or recent reperfusion therapy [147]. Risk stratification tools that integrate clinical factors, imaging findings, and biomarkers may aid in identifying patients who can safely receive anticoagulation [148]. In practice, close neurological monitoring and repeat imaging are essential when initiating or intensifying anticoagulant therapy. Evidence guiding bleeding risk assessment in the context of DVT prophylaxis is derived from both stroke-specific observational studies and extrapolated data from general medical populations.

### 6.7. Evidence Gaps and Future Directions

This review highlights several critical evidence gaps in pharmacological DVT prophylaxis for AIS patients with lower limb paralysis. First, dedicated randomized controlled trials evaluating extended prophylaxis specifically in AIS populations are urgently needed to validate the efficacy and safety of this approach. Second, the role of DOACs for primary DVT prevention in acute AIS remains undefined and requires prospective investigation. Third, the optimal timing of anticoagulation initiation after reperfusion therapies (particularly mechanical thrombectomy) lacks high-quality evidence and warrants further study. Fourth, head-to-head comparisons of different LMWH agents and dosing strategies in AIS patients are limited. Addressing these gaps through well-designed clinical trials will be essential to refining evidence-based recommendations for this high-risk population.

## 7. Mechanical Prophylaxis for DVT Prevention in AIS Patients with Lower Limb Paralysis

**Figure 2:**
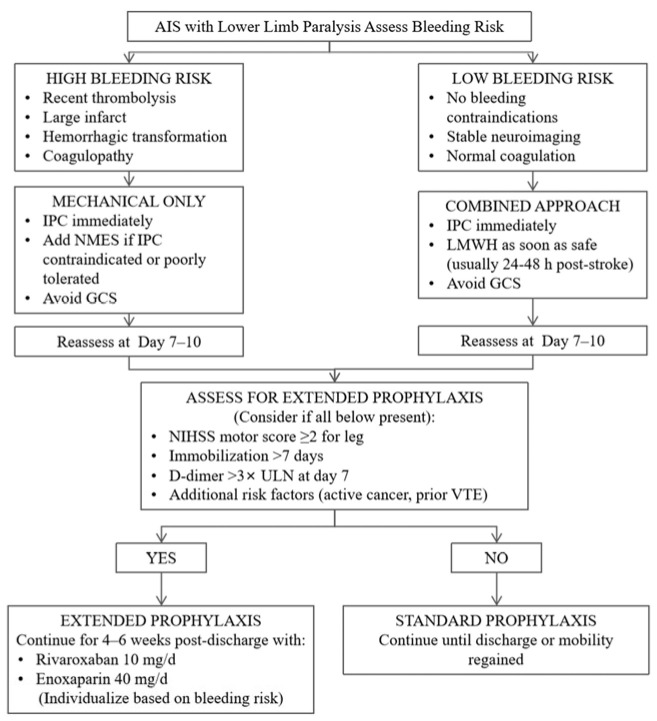
Proposed algorithm for DVT prevention in AIS patients with lower limb paralysis.

Mechanical prophylaxis represents a critical component of DVT prevention in acute ischemic stroke patients with lower limb paralysis, particularly for individuals with contraindications to pharmacological anticoagulation or a high bleeding risk. By enhancing venous return and reducing venous stasis, mechanical methods directly address one of the central elements of Virchow’s triad. In Figure 2, a flowchart detailing the prophylaxis protocol for deep vein thrombosis in patients with lower limb paralysis due to ischemic stroke is presented.

### 7.1. Intermittent Pneumatic Compression (IPC)

Intermittent pneumatic compression (IPC) devices are the most extensively studied mechanical modality in stroke populations [149,150]. IPC functions by rhythmically inflating and deflating cuffs placed around the lower limbs, thereby simulating the physiological muscle pump and increasing venous blood flow velocity.

Large randomized controlled trials over the past decade have demonstrated that IPC significantly reduces the incidence of proximal DVT in immobile stroke patients [4]. The landmark CLOTS-3 trial showed a relative risk reduction for DVT when IPC was applied early and consistently [151,152]. Subsequent real-world studies and meta-analyses confirmed these findings, particularly in patients with severe motor deficits and prolonged immobility. Importantly, IPC is associated with minimal bleeding risk, making it especially valuable in patients with hemorrhagic transformation risk or recent thrombolysis. However, adherence remains a challenge. Discomfort, skin irritation, and device interruptions may reduce effective usage time. Education of nursing staff and patients, along with regular skin inspection, is essential to maximize benefit.

### 7.2. Graduated Compression Stockings (GCS)

Graduated compression stockings were historically used for DVT prevention; however, evidence accumulated over the last decade has demonstrated limited efficacy in stroke patients. Multiple trials have failed to show a significant reduction in DVT incidence with GCS use, and concerns regarding skin breakdown, ischemia, and poor tolerance have further limited their role [153,154,155]. Therefore, patients with AIS should also avoid the use of GCS, particularly those with sensory impairment or peripheral arterial disease. In selected cases where IPC is unavailable, GCS may be considered with caution, but they should not replace IPC as first-line mechanical prophylaxis [155].

### 7.3. Combined Mechanical and Pharmacological Strategies

Increasing evidence supports the synergistic use of mechanical and pharmacological prophylaxis [156]. Combining IPC with low-molecular-weight heparin has been shown to further reduce DVT incidence compared with either modality alone, without a proportional increase in bleeding risk [157]. For patients with lower limb paralysis who remain immobile beyond the acute phase, this combined approach may hold particular clinical relevance as a targeted therapeutic strategy. Table 2 provides a comprehensive comparison of pharmacological, mechanical, and rehabilitative prophylactic strategies for DVT prevention in AIS patients.

## 8. Early Rehabilitation and Mobilization as a Cornerstone of DVT Prevention

Beyond pharmacological and mechanical measures, early rehabilitation and mobilization play a pivotal role in preventing DVT while simultaneously promoting neurological recovery. Lower limb paralysis directly impairs venous return, and restoring even partial muscle activity can substantially reduce venous stasis.

### 8.1. Timing and Safety of Early Mobilization

Early mobilization within 24 h after AIS has been shown to be safe in carefully selected patients [158,159]. While ultra-early intensive mobilization may not improve functional outcomes in all cases, gradual and structured mobilization strategies are associated with reduced medical complications, including DVT. Passive range-of-motion exercises, bedside sitting, and assisted standing can be initiated even in patients with severe paralysis [160,161]. These interventions stimulate venous flow, prevent muscle atrophy, and improve endothelial function. Importantly, mobilization protocols must be individualized based on stroke severity, hemodynamic stability, and hemorrhagic risk.

### 8.2. Neuromuscular Electrical Stimulation

Neuromuscular electrical stimulation (NMES) has emerged as an adjunctive tool for DVT prevention in patients unable to voluntarily activate lower limb muscles [162]. NMES induces rhythmic muscle contractions, mimicking the physiological muscle pump. Randomized trials have demonstrated that NMES increases venous blood flow velocity and reduces calf vein diameter, markers associated with decreased thrombosis risk [163,164]. When combined with IPC or anticoagulation, NMES may offer additional protective effects, particularly in patients with profound paralysis or prolonged immobilization [165].

### 8.3. Rehabilitation Intensity and Long-Term Protection

Sustained rehabilitation engagement throughout hospitalization and early post-discharge periods is essential. Patients who transition rapidly from bed rest to ambulation demonstrate significantly lower rates of DVT and pulmonary embolism [166]. Rehabilitation intensity should progressively increase as neurological recovery permits, reinforcing the concept that DVT prevention is a dynamic, ongoing process rather than a one-time intervention.

## 9. Multidisciplinary and Integrated Management Approaches

Optimal DVT prevention in AIS patients with lower limb paralysis requires a coordinated, multidisciplinary approach. Neurologists, rehabilitation physicians, nurses, hematologists, and physical therapists must collaborate to balance thrombotic and bleeding risks while promoting recovery.

### 9.1. Stroke Unit-Based Protocols

Dedicated stroke units with standardized DVT prevention protocols have consistently demonstrated lower complication rates and improved outcomes [167]. These protocols typically integrate early risk stratification, prompt initiation of IPC, timely anticoagulation, and early rehabilitation. Standardization reduces variability in care delivery and ensures that high-risk patients do not experience delays in prophylaxis. Importantly, protocols should be flexible enough to accommodate individualized clinical judgment.

### 9.2. Nursing-Led Surveillance and Education

Nurses play a central role in DVT prevention through daily risk reassessment, device management, and patient education [168]. Regular limb inspection, monitoring of skin integrity, and ensuring compliance with IPC significantly influence prophylactic effectiveness. Patient and caregiver education is equally critical, particularly during transitions of care. Awareness of DVT symptoms and adherence to prescribed prophylactic measures after discharge can prevent late thromboembolic events.

## 10. Challenges, Knowledge Gaps, and Future Directions

Despite substantial progress, several challenges remain in DVT prevention for AIS patients with lower limb paralysis. First, the optimal duration of prophylaxis remains uncertain. While extended prophylaxis shows promise, stroke-specific randomized trials are limited. Second, the integration of biomarkers and predictive models into routine practice is still evolving. Third, emerging therapies targeting immunothrombosis, neutrophil extracellular traps, and endothelial dysfunction require further clinical validation.

Future research should prioritize large, stroke-specific trials of extended anticoagulation and validation of AI-based risk prediction tools. Exploration of personalized prophylaxis based on genetic and biomarker profiles and the development of wearable technologies to monitor mobility and venous flow in real time are also needed.

## 11. Conclusions

Acute ischemic stroke patients with lower limb paralysis represent a uniquely vulnerable population with a markedly elevated risk of deep vein thrombosis. The pathogenesis of DVT in this group is multifactorial, encompassing venous stasis due to immobility, endothelial injury, hypercoagulability, and systemic inflammation.

Over the past decade, significant advances have been made in understanding these mechanisms and translating them into effective preventive strategies. Pharmacological anticoagulation, mechanical prophylaxis, early rehabilitation, and multidisciplinary care form the foundation of contemporary DVT prevention. Increasingly, personalized approaches that integrate clinical risk factors, biomarkers, and dynamic assessment are shaping future practice.

In conclusion, optimal prevention of DVT in AIS patients with lower limb paralysis requires a comprehensive, individualized, and evolving strategy. Continued interdisciplinary collaboration and high-quality research are essential to refining prevention protocols, reducing thromboembolic complications, and ultimately improving functional recovery and survival in this high-risk population.

## Figures and Tables

**Table 1:** Summary of DVT risk factors specific to AIS patients with lower limb paralysis.

Category	Risk Factors	Clinical Relevance
Patient-Related	Advanced age	Reduced physiological reserve, increased comorbidity burden
	Prior history of VTE	Significantly elevated recurrence risk
	Active malignancy	Cancer-associated hypercoagulable state
	Obesity	Impaired venous return, chronic proinflammatory state
	Heart failure	Venous congestion, reduced mobility
Stroke-Related	High NIHSS score (especially motor score ≥ 2 for leg)	Severe motor impairment, prolonged immobility
	Lower limb paralysis/hemiplegia	Complete loss of calf muscle pump function
	Large infarct volume	Extended immobilization, autonomic dysregulation
	Hemorrhagic transformation	Delayed or withheld anticoagulation
Complication-Related	Acute infections (pneumonia, UTI)	Systemic inflammation, immunothrombosis activation
	Dehydration	Hemoconcentration, increased blood viscosity
	Prolonged immobilization (>7 days)	Sustained venous stasis
	Post-stroke autonomic dysfunction	Impaired venous tone and hemodynamics

**Table 2:** Comparison of prophylactic strategies for DVT prevention in AIS patients.

Strategy	Modality	Mechanism of Action	Indications	Contraindications/Limitations	Evidence Level
Pharmacological	Low-Molecular-Weight Heparin (LMWH)	Inhibition of factor Xa and IIa	Standard prophylaxis for immobilized AIS patients	Active bleeding, hemorrhagic transformation, coagulopathy	High (stroke-specific RCTs)
	Unfractionated Heparin (UFH)	Inhibition of factor IIa and Xa	Alternative when LMWH unavailable	Requires monitoring, higher HIT risk	Moderate
	Direct Oral Anticoagulants (DOACs)	Factor Xa inhibition (rivaroxaban, betrixaban)	Extended prophylaxis post-discharge in selected high-risk patients	Limited stroke-specific data, bleeding risk	Moderate (extrapolated from medically ill patients)
Mechanical	Intermittent Pneumatic Compression (IPC)	Simulates calf muscle pump, increases venous return	All immobilized AIS patients, especially those with bleeding contraindications	Peripheral arterial disease, severe leg trauma, skin breakdown	High (CLOTS-3 trial)
	Graduated Compression Stockings (GCS)	Graduated external compression	Not recommended as first-line prophylaxis	Limited efficacy in stroke, skin ischemia risk, poor tolerance	Low (evidence against routine use)
	Neuromuscular Electrical Stimulation (NMES)	Electrical stimulation induces rhythmic muscle contractions	Adjunct in patients unable to voluntarily activate leg muscles	Device availability, patient tolerance, limited large-scale trials	Moderate (emerging evidence)
Rehabilitative	Early mobilization	Restores physiological muscle pump, reduces venous stasis	As soon as medically safe (typically within 24–48 h)	Hemodynamic instability, severe neurological deficit, malignant edema	Moderate
	Passive range-of-motion exercises	Maintains joint mobility, stimulates venous flow	Patients with complete paralysis unable to participate actively	None significant	Low–Moderate
	Assisted standing/ambulation	Weight-bearing activates the venous pump, improves venous return	As neurological recovery permits, during the rehabilitation phase	Fall risk, orthostatic hypotension, fatigue	Moderate

## Data Availability

No new data were created or analyzed in this study. Data sharing is not applicable to this article.

## Abbreviations

The following abbreviations are used in this manuscript:

AIS	Acute Ischemic Stroke
ANS	Autonomic Nervous System
CRP	C-reactive Protein
DOACs	Direct Oral Anticoagulants
DVT	Deep Vein Thrombosis
eNOS	Endothelial Nitric Oxide Synthase
GCS	Graduated Compression Stockings
ICAM-1	Intercellular Adhesion Molecule-1
IPC	Intermittent Pneumatic Compression
LMWH	Low-Molecular-Weight Heparin
NETs	Neutrophil Extracellular Traps
NIHSS	National Institutes of Health Stroke Scale
NLR	Neutrophil-to-Lymphocyte Ratio
NMES	Neuromuscular Electrical Stimulation
PAI-1	Plasminogen Activator Inhibitor-1
PE	Pulmonary Embolism
PPARγ	Peroxisome Proliferator-Activated Receptor Gamma
ROS	Reactive Oxygen Species
tPA	Tissue Plasminogen Activator
UFH	Unfractionated Heparin
VCAM-1	Vascular Cell Adhesion Molecule-1
VTE	Venous Thromboembolism
vWF	von Willebrand Factor
